# Psychometric properties of the Turkish version of the Flexible or Rigid Control of Eating Scale in women

**DOI:** 10.3389/fpsyg.2026.1826677

**Published:** 2026-09-17

**Authors:** Çağdaş Salih Meriç, Mustafa Özgür, Emre Duman, Hacı Ömer Yılmaz

**Affiliations:** 1 Department of Nutrition and Dietetics, Faculty of Health Sciences, Gaziantep University, Gaziantep, Türkiye; 2 Department of Nutrition and Dietetics, Faculty of Health Sciences, Burdur Mehmet Akif Ersoy University, Burdur, Türkiye; 3 Department of Nutrition and Dietetics, Faculty of Health Sciences, Siirt University, Siirt, Türkiye; 4 Department of Nutrition and Dietetics, Faculty of Health Sciences, Bandırma Onyedi Eylül University, Balıkesir, Türkiye

**Keywords:** eating control, eating disorders, flexible control, psychometric validation, rigid control, Turkish adaptation

## Abstract

**Background:**

Eating control is a central construct in eating disorder research; however, existing measures often insufficiently capture its multidimensional nature and rarely distinguish rigid and flexible regulatory styles. The Flexible or Rigid Control of Eating Scale (FORCES) was developed to address this limitation; however, no culturally adapted instrument assessing rigid and flexible eating control has previously been validated in Turkish populations.

**Methods:**

This study aimed to translate, culturally adapt, and psychometrically evaluate the FORCES in a non-clinical sample of Turkish women. Two independent cross-sectional online samples were included. Exploratory factor analysis (EFA; *n* = 416) was conducted to examine whether the original multidimensional structure would be replicated in a Turkish context, followed by confirmatory factor analysis (CFA; *n* = 417). Internal consistency, test–retest reliability, convergent validity, construct validity, and Differential Item Functioning (DIF) were evaluated. Item Response Theory (IRT)-based Rasch analyses examined item-level psychometric properties. Construct validity was assessed using the Three-Factor Eating Questionnaire (TFEQ-R21), Dichotomous Thinking in Eating Disorders Scale (DTEDS), State–Trait Anxiety Inventory (STAI), and Body Mass Index (BMI). Test–retest reliability was evaluated in a subsample (*n* = 53) over a two-week interval.

**Results:**

EFA supported the original five-factor structure, explaining 62.85% of the variance, with factor loadings ranging from 0.589 to 0.921. CFA confirmed this structure and demonstrated good model fit (χ^2^/df = 2.655, CFI = 0.951, TLI = 0.940, RMSEA = 0.063, SRMR = 0.035). The overall scale demonstrated high internal consistency (α = 0.874; ω = 0.880), while subscale reliability coefficients ranged from 0.766 to 0.917 for Cronbach’s alpha and from 0.775 to 0.918 for McDonald’s omega. Rasch analyses supported satisfactory item functioning and substantial measurement equivalence across BMI groups. Test–retest reliability coefficients indicated satisfactory temporal stability (ICC = 0.726–0.821). Rigid control dimensions were moderately associated with emotional eating, dichotomous thinking, anxiety, and BMI (*r* = 0.20–0.49, *p* < 0.01), whereas flexible control dimensions showed weaker but significant associations (*r* = 0.10–0.25, *p* < 0.01), supporting the distinction between rigid and flexible eating control.

**Conclusion:**

The Turkish version of the FORCES is a reliable and valid instrument for assessing flexible and rigid eating control in women. By distinguishing maladaptive rigid control from adaptive flexible regulation, the scale provides a multidimensional framework for investigating eating regulation and may support future cross-cultural and behavioral research in Turkish populations.

## 1 Background

Eating behavior is shaped by complex interactions among cognitive regulation, emotional processes, and learned behavioral patterns that influence how individuals manage food intake in daily life. Within this framework, eating control has emerged as a central construct in research on dietary behavior, weight regulation, and eating-related psychopathology (Chawner and Filippetti, 2024; Dicu et al., 2024). Traditionally, eating control has been conceptualized primarily through dietary restraint, whereby stronger restriction was assumed to reflect greater self-regulatory capacity (Russell and Russell, 2020). However, growing evidence suggests that this unidimensional perspective fails to capture important qualitative differences in how individuals regulate eating behavior, differences that may have distinct psychological and behavioral consequences (Bandelin-Franke et al., 2023; Dakin et al., 2024).

Contemporary theoretical models distinguish between rigid and flexible forms of eating control. Rigid control is characterized by strict dietary rules, dichotomous thinking, heightened vigilance regarding food intake, and negative emotional responses following perceived dietary lapses (Nicholls et al., 2025). This style of regulation has consistently been associated with maladaptive outcomes, including emotional eating, disinhibition, anxiety, and increased vulnerability to eating pathology (Bagrowska and Gawęda, 2023; Nader et al., 2025). In contrast, flexible control reflects a more adaptive approach involving moderation, contextual decision-making, and tolerance of dietary deviations, and has been linked to better psychological well-being and more sustainable eating behaviors (Brytek-Matera and Han, 2021; Modrzejewska et al., 2025). Importantly, rigid and flexible control are not considered opposite ends of a single continuum, but rather distinct and potentially coexisting regulatory processes that may differentially influence eating-related outcomes (Nicholls et al., 2025).

Despite the theoretical relevance of this distinction, commonly used eating behavior measures do not adequately capture the multidimensional nature of eating control. Widely used instruments such as the Three-Factor Eating Questionnaire (TFEQ-R21) primarily assess cognitive restraint and provide limited differentiation between adaptive and maladaptive forms of regulation (Duarte et al., 2020). As a result, emotionally and cognitively relevant processes, including worry, anxiety, and dichotomous thinking, are often underrepresented within existing assessment frameworks. This limitation restricts a more nuanced understanding of how different regulatory styles contribute to eating-related psychopathology and weight-related outcomes (Ha and Lim, 2023; Rossi et al., 2025).

The Flexible or Rigid Control of Eating Scale (FORCES) was developed to address this measurement gap by conceptualizing eating control as a multidimensional construct rather than a unidimensional form of dietary restraint. The original development and validation of the FORCES was conducted across three independent samples of women and supported a theoretically grounded five-factor structure representing distinct rigid and flexible eating control processes. Consistent with the broader eating behavior literature, the initial validation studies focused exclusively on women, reflecting the heightened salience of eating control, body image concerns, and dieting behaviors among women. Psychometric evaluations in the original studies primarily employed exploratory and confirmatory factor analytic approaches and demonstrated satisfactory internal consistency and construct validity in community-based samples of women. To date, psychometric evaluation of the FORCES has remained limited, and no validated adaptation has previously been available in Turkish or other non-Western populations.

The FORCES comprises five theoretically derived subdimensions (“Strict Behaviors,” “Negative Emotions,” “Worry,” “Flexible Beliefs,” and “Positive Emotions”), which are hierarchically organized under the higher-order constructs of “Rigid Control” and “Flexible Control.” Initial validation findings demonstrated meaningful associations with emotional eating, cognitive restraint, and indicators of eating-related psychopathology, supporting both the construct validity and clinical relevance of the instrument (de Pádua Constâncio et al., 2025; Meule et al., 2025; Péneau et al., 2022). By simultaneously capturing behavioral, emotional, and cognitive components of eating regulation, the FORCES provides a more comprehensive assessment framework than traditional restraint-based measures.

Conceptually, the FORCES is grounded in a multidimensional model of eating regulation that distinguishes qualitatively different control strategies rather than positioning eating restraint along a single continuum. Within this framework, rigid control reflects a rule-based and dichotomous approach to eating characterized by inflexible dietary boundaries, heightened vigilance, and increased sensitivity to perceived dietary violations, patterns consistently associated with maladaptive outcomes such as disinhibition, binge eating, and weight cycling.

In contrast, flexible control represents a more adaptive regulatory style involving moderation, contextual decision-making, and tolerance of dietary deviations, and has been linked to more sustainable eating behaviors and better psychological well-being. To adequately capture these multidimensional regulatory processes, psychometrically robust assessment tools are required. Importantly, these regulatory styles are conceptualized as distinct yet potentially co-occurring processes that may differentially contribute to eating-related outcomes and obesity-related risk (Nicholls et al., 2025).

Despite these promising findings, the psychometric properties of the FORCES have not previously been evaluated in Turkish populations, and culturally adapted multidimensional instruments capable of differentiating rigid and flexible eating control remain limited in Türkiye. Given the sociocultural variability in eating-related beliefs, body image norms, and dietary practices, cross-cultural psychometric evaluation of the FORCES represents an important step toward improving the assessment of eating regulation within non-Western populations.

The psychological correlates of eating control extend beyond eating-specific behaviors to broader affective and cognitive processes. Anxiety, particularly, appears closely linked to rigid regulation patterns. State anxiety reflects transient emotional responses to situational stressors, whereas trait anxiety represents a more stable predisposition toward anxiety across contexts (Moszak et al., 2025; Quevedo-Bayona et al., 2025). Elevated anxiety has been associated with increased dietary worry, strict rule adherence, and emotional eating behaviors (Valente et al., 2025; Varallo et al., 2025). Similarly, dichotomous thinking constitutes a central cognitive mechanism underlying rigid eating control (Levinson et al., 2025). Individuals who evaluate eating behavior in black-and-white terms are more likely to interpret dietary lapses as personal failures, thereby reinforcing maladaptive emotional and behavioral responses. Consequently, measures such as the State–Trait Anxiety Inventory (STAI) and the Dichotomous Thinking in Eating Disorders Scale (DTEDS) provide theoretically relevant criteria for evaluating the construct validity of multidimensional eating control instruments (Kikuchi et al., 2024).

Cross-cultural validation of psychometric instruments is essential because eating-related beliefs, emotional expression, and self-regulatory norms are shaped by sociocultural context (Fekar Gharamaleki and Fathipour-Azar, 2024; Gultekin and Bozkir, 2025). Cultural factors may influence how rigid and flexible eating control strategies are experienced, expressed, and socially reinforced. In Türkiye, traditional dietary practices increasingly coexist with rapid changes in food environments, heightened exposure to dieting culture, and growing sociocultural emphasis on body image, particularly among young women (Çınaroğlu and Yılmazer, 2025; Dikyol Mutlu et al., 2022; Pehlivan et al., 2025). These sociocultural dynamics may shape both adaptive and maladaptive forms of eating regulation and highlight the importance of culturally sensitive assessment tools.

Although the original FORCES validation studies focused exclusively on women, consistent with the predominance of women in eating control and body image research (Braden et al., 2025; Limiñana-Gras et al., 2026; Nicholls et al., 2025), no culturally adapted multidimensional instrument capable of distinguishing rigid and flexible eating control has previously been validated in Turkish populations. Given the absence of prior multidimensional assessments of eating control in Turkish women, establishing the psychometric properties of the FORCES in this population represents an important preliminary step before broader validation across sex and clinical groups. Moreover, maintaining consistency with the original validation framework supports more direct cross-cultural comparison of the psychometric structure and theoretical interpretation of the scale.

Accordingly, the present study aimed to translate, culturally adapt, and psychometrically evaluate the Turkish version of the FORCES in women in Türkiye. By providing a culturally grounded and multidimensional assessment tool, this study seeks to advance the evaluation of eating regulation beyond traditional unidimensional restraint models and to facilitate future cross-cultural, clinical, and behavioral research on adaptive and maladaptive eating control processes.

## 2 Methods

### 2.1 Validation, translation and cultural adaptation

The translation and cultural adaptation of the FORCES into Turkish were conducted in accordance with internationally accepted guidelines for cross-cultural adaptation of self-report measures (Beaton et al., 2000), with the aim of achieving semantic, idiomatic, experiential, and conceptual equivalence. In the first stage, the original English version of the scale was independently translated into Turkish by a native Turkish speaker fluent in English and knowledgeable about eating behavior research. Subsequently, a bilingual translator whose native language was English and who was blinded to the original scale performed a back-translation of the Turkish version into English. The original version and the back-translated version were systematically compared to identify discrepancies in meaning, wording, and conceptual content. Based on this comparison, necessary revisions were made to the Turkish translation to improve clarity and conceptual alignment. Following these revisions, a second back-translation was carried out to ensure consistency and accuracy. Consensus on the final Turkish version was achieved through detailed discussions among the research team and the translators. Additionally, an independent panel of eight external experts in nutrition and dietetics, who were not part of the research team and had no prior involvement with the scale, evaluated the translated items during the content validity phase. Particular attention was given to ensuring that the translated items achieved not only linguistic accuracy but also cultural appropriateness for Turkish women. During the cultural adaptation phase, each FORCES item was systematically evaluated for semantic, conceptual, and experiential equivalence, with careful consideration of Turkish idiomatic expressions and sociocultural norms related to eating regulation.

For example, strict control statements such as “I follow strict rules about how much I can eat” were rendered using phrasing that reflects culturally familiar notions of self-imposed dietary discipline in Türkiye (e.g., “Ne kadar yiyebileceğim konusunda katı kurallarım vardır”), thereby maintaining the construct of rigid regulation while ensuring natural language use. Similarly, emotional reaction items such as “If I eat something that I would normally not allow myself to eat, I feel tense, irritable, and/or depressed” were translated in a manner that preserved the affective intensity and self-evaluative nuance inherent in the original wording. Because expressions of self-evaluation and emotional distress may vary across cultural contexts, linguistically natural and culturally meaningful equivalents were selected. For instance, the item was adapted as “Normalde kendime yeme izni vermediğim bir şeyi yediğimde, gergin, sinirli ve/veya depresif hissederim” retaining the emotional tone and the internally directed response central to rigid eating control.

The measurement properties were operationalized according to the Consensus-based Standards for the Selection of Health Status Measurement Instruments (COSMIN), and reporting followed the Strengthening the Reporting of Observational Studies in Epidemiology (STROBE) guidelines.

#### 2.1.1 Content validity

Following translation and cultural adaptation, content validity was assessed in line with the recommendations of Polit and Beck (2006). A panel of eight experts with expertise in nutrition and dietetics evaluated the relevance of each item using a four-point rating scale (1 = not relevant, 2 = somewhat relevant, 3 = quite relevant, 4 = highly relevant). The item-level content validity index (I-CVI) was calculated as the proportion of experts assigning ratings of 3 or 4, with values of ≥0.78 considered acceptable. The scale-level content validity index (S-CVI/Ave) was computed as the mean of all I-CVI values, and S-CVI/UA and modified kappa (k*) statistics were also examined to adjust for chance agreement. The results indicated excellent content validity, with an S-CVI/Ave of 0.99, reflecting a high level of expert agreement on item relevance and conceptual adequacy (Polit et al., 2007). Overall, these findings demonstrate that the Turkish version of the scale possesses strong content validity and is suitable for further psychometric evaluation.

#### 2.1.2 Face validity

To further evaluate the cultural relevance and comprehensibility of the translated scale, face validity was assessed using a pilot group of 20 volunteer participants. Individual interviews were conducted with each participant to explore their understanding of the items and to identify any ambiguous or culturally inappropriate expressions. During pilot testing, minor wording revisions were made to Items 4, 9, and 16 to improve clarity. Participants reported that the original literal translations of these items sounded overly formal. Therefore, lexical simplifications were implemented to enhance comprehensibility while preserving conceptual meaning. No items were removed. Feedback obtained during this stage was used to refine specific lexical and syntactic elements, resulting in the final Turkish version of the FORCES. To enhance transparency and facilitate future cross-cultural and psychometric research, the finalized Turkish version of the FORCES is provided in Supplementary Table S1.

### 2.2 Psychometric testing

#### 2.2.1 Study design

Permission to translate and validate the FORCES was obtained from the authors of the original validation study. The research was conducted as two independent cross-sectional studies administered online, with a total sample of 833 women. Both Study 1 and Study 2 were completed online. To enhance data integrity and minimize the risk of duplicate submissions, the survey platform was configured to restrict multiple entries from the same device/IP address, and response datasets were screened for potential duplication based on completion time, response patterns, and internal consistency across key variables. The study protocol was approved by the Gaziantep Islamic Science and Technology University Non-Interventional Clinical Research Ethics Committee (Protocol No: 2025/642 and Decision No: 642.48.16) and adhered to the Declaration of Helsinki. Electronic informed consent was obtained by asking all participants to click a button at the beginning of the online survey indicating their acceptance of participation. Participation was voluntary and anonymous, and no payment or reward was offered to participants.

#### 2.2.2 Participants

Recruitment was conducted independently for each study using separate participant samples. Previous methodological recommendations suggest that an adequate sample size for scale validation ranges between five and twenty participants per item (Alpar, 2018). In this cross-sectional study, data from 416 women in the first stage and 417 women in the second stage were used to evaluate the validity and reliability of the scale. In addition to participant-to-item ratio recommendations, sample size adequacy was evaluated in relation to statistical power considerations. Given the total sample size (*N* = 833), the study was sufficiently powered to detect small-to-moderate effect sizes (e.g., *r* ≈ 0.10–0.20) with high statistical power (β > 0.90) in correlational analyses. Furthermore, the sample size exceeds commonly recommended thresholds for obtaining stable and replicable factor solutions in both exploratory and confirmatory factor analyses.

Eligibility criteria included being women, having no diagnosed chronic disease, physical disability, pregnancy, or breastfeeding status that could influence eating behavior or body weight regulation, possessing adequate Turkish reading and comprehension skills, and providing informed consent. Individuals with food allergies, acute or chronic conditions affecting dietary intake, disease-specific dietary regimens, taste or smell disorders, or those who did not provide informed consent were excluded. Participants were not formally screened for eating disorders or psychiatric diagnoses such as depression or anxiety disorders, nor for the use of psychotropic medications. However, individuals reporting chronic medical conditions, disease-specific dietary regimens, or health conditions known to affect eating behavior were excluded based on self-report. Therefore, while the sample represents a generally healthy, non-clinical population, the presence of undiagnosed or subclinical psychological conditions cannot be entirely ruled out. Participants were recruited by distributing the questionnaire link through social media platforms. The online questionnaire included a detailed explanation of the study objectives and procedures, followed by an informed consent section that required active confirmation before participation.

### 2.3 Data collection tools

The demographic form contains information such as the participants’ age, marital status, educational levels, body weight and height. Anthropometric measurements (weight and height) were assessed based on the self-reports of the participants. BMI was calculated by dividing body weight by the square of height. Participants were classified according to BMI as underweight (<18.5 kg/m^2^), normal weight (18.5–24.99 kg/m^2^), overweight (25.0–29.99 kg/m^2^), or having obesity (≥30.0 kg/m^2^) (World Health Organization, 2000).

#### 2.3.1 The Flexible or Rigid Control of Eating Scale

The FORCES was developed by Nicholls et al. (2025) and consists of a 19-item self-report instrument developed to assess individual differences in cognitive and behavioral patterns related to dietary control, specifically distinguishing between rigid and flexible styles of eating regulation. The scale was derived through a comprehensive item-generation process that included expert consultation and theoretical grounding in the literature on dietary restraint and disinhibited eating, followed by rigorous psychometric evaluation across three independent samples of women. The development of the FORCES began with an initial pool of 43 items designed to capture a broad range of behaviors and experiences associated with dietary control, including behavioral efforts (e.g., time and energy devoted to dieting), cognitive beliefs, and emotional responses to perceived diet “violations.” Exploratory and confirmatory factor analyses were employed to refine the item pool and identify the underlying factorial structure, resulting in a final measure comprising five distinct factors: Strict Behaviors (5 items), Negative Emotions (5 items), Worry (3 items), Flexible Beliefs (3 items), and Positive Emotions (3 items). These subscales are grouped into two higher-order constructs: “*Rigid Control*” computed as the sum of “Strict Behaviors,” “Negative Emotions,” and “Worry” (13 items), and “*Flexible Control*” computed as the sum of “Flexible Beliefs” and “Positive Emotions” (6 items). All items in the FORCES are rated on a four-point Likert scale ranging from “1 = Definitely not true” to “4 = Definitely true” (1 = Definitely not true, 2 = Not true, 3 = True, 4 = Definitely true) and the final factor structure demonstrated good internal consistency and replicability across multiple samples. This clear separation between rigid and flexible control contributes to improved construct validity relative to older measures that often conflated these dimensions (Nicholls et al., 2025).

#### 2.3.2 Dichotomous thinking in eating disorders scale

The DTEDS was developed by Byrne et al. (2008) and consists of 11 items, and participants receive a score ranging from 1 to 4 for each item based on their responses. Total scores obtained from the subscales are divided by the number of items within each subscale to calculate mean scores. Subsequently, the overall score, ranging from 1 to 4, is obtained by calculating the average of the two subscale scores. As this score exceeds 2, it is concluded that individuals may exhibit dichotomous thinking disorder and, accordingly, a related eating disorder (Byrne et al., 2008). The Turkish validity and reliability of the DTEDS were established by Kütahya et al. (2022), and Cronbach’s alpha was reported as 0.808 (Kütahya et al., 2022).

#### 2.3.3 State–trait anxiety inventory

The STAI was developed by Spielberger et al. (1971) inspired by the two-factor anxiety theory to determine state and trait anxiety levels separately. The State–Trait Anxiety Inventory (STAI-S and STAI-T) is a self-report questionnaire consisting of brief expressions. It comprises two subscales of 20 items, forty items in total (Spielberger et al., 1971). Development of the short version of the STAIS-5 and STAIT-5 conducted by Zsido et al. (2020). The Turkish validity and reliability study was conducted by Döner et al. (2024). The Cronbach alpha coefficients of the State and Trait Anxiety Inventory Short Version (STAIS-5 and STAIT-5) were calculated as 0.838 and 0.837, respectively. The short form is a 4-point Likert-type inventory consisting of 10 items in total, 5 for state anxiety and 5 for trait anxiety. Direct and inverted statements are added together by weighing separately. The higher scores indicate higher anxiety levels (Döner et al., 2024).

#### 2.3.4 Three-factor eating questionnaire

The TFEQ-R21 was originally developed by Stunkard and Messick (1985) to assess the behavioral and cognitive components of eating (Stunkard and Messick, 1985). The Turkish adaptation was validated by Karakuş et al. (2016), and Cronbach’s alpha coefficients ranged between 0.787 and 0.870 across subscales (Karakuş et al., 2016). The scale comprises 21 items rated on a four-point Likert scale and includes three subscales: “*Uncontrolled Eating*,” “*Cognitive Restraint*,” and “*Emotional Eating*.” Higher scores on each subscale reflect greater levels of the corresponding eating behavior.

### 2.4 Statistical analysis (Study 1 and Study 2)

Data analyses were carried out separately for Study 1 and Study 2. Prior to factor analytic procedures, the suitability of the dataset for factor analysis was examined using the Kaiser-Meyer-Olkin (KMO) measure of sampling adequacy and Bartlett’s test of sphericity. Preliminary screening indicated that the dataset contained no missing values. Although the original FORCES structure has previously been established, Exploratory Factor Analysis (EFA) was conducted to evaluate whether the multidimensional structure of the scale would be replicated within a different linguistic and sociocultural context, consistent with recommendations for cross-cultural psychometric adaptation and validation (Callegaro Borsa et al., 2012; Watkins, 2018).

In Study 1, data obtained from 416 participants were subjected to EFA using SPSS version 27.0. The principal axis factoring extraction method was selected to identify the latent structure underlying the scale items. Considering the conceptual relatedness of the subdimensions, a direct oblimin rotation was applied. The decision regarding the number of factors was based on a combination of eigenvalues, visual inspection of the scree plot, and theoretical interpretability. Consistent with psychometric adaptation guidelines, EFA and CFA were sequentially performed in independent samples to evaluate both exploratory and confirmatory structural validity. To verify the factor structure derived from EFA, CFA was conducted in Study 2 on an independent sample of 417 participants. The hypothesized first-order correlated five-factor model was compared with a second-order hierarchical model representing the higher-order Rigid Control and Flexible Control dimensions. Because the data met assumptions of multivariate normality, CFA was performed using the maximum likelihood estimation method. Model testing was conducted with AMOS version 24, and model adequacy was evaluated using multiple goodness-of-fit indices, including the Chi-square/Degree of Freedom (χ^2^/df) ratio, Standardized Root Mean Square Residual (SRMR), Root Mean Square Error of Approximation (RMSEA), Comparative Fit Index (CFI), Incremental Fit Index (IFI), Tucker Lewis Index (TLI), Goodness of Fit Index (GFI), and Adjusted Goodness of Fit Index (AGFI), in accordance with commonly accepted cut-off criteria (Hooper et al., 2008; Munro, 2005; Schumacker and Lomax, 2010; Şimşek, 2020; Waltz et al., 2010).

The internal consistency of the scale and its subdimensions was evaluated using Cronbach’s alpha and McDonald’s omega (ω) coefficients. Convergent validity was further evaluated by calculating the Average Variance Extracted (AVE) and Composite Reliability (CR). The AVE and CR were calculated based on the standardized factor loadings and error variances obtained from the CFA model. Moving beyond Classical Test Theory, modern psychometric evaluations were conducted using Item Response Theory (IRT), specifically the Rasch model, to assess item-level properties and measurement invariance. Model fit was evaluated using Infit and Outfit mean-square (MnSq) statistics (acceptable range: 0.5–1.5), and overall test precision was indicated by Person Reliability and MADaQ3 statistics. Person-Item maps (Wright maps) were generated to visually evaluate the alignment between participant trait levels and item difficulty thresholds. Additionally, to address measurement invariance across BMI categories (participants classified as underweight or normal weight and participants classified as having overweight or obesity), Differential Item Functioning (DIF) was tested. A non-significant adjusted *p*-value (>0.05) indicates that the item functions equivalently across different BMI groups without bias. The snowIRT and snow RMM module in Jamovi software (Version 5.1) was used to conduct the Rasch modeling, including item fit evaluations, person reliability estimation, and DIF calculations across BMI categories (Seol, 2025).

The temporal stability of the scale was examined through test–retest reliability analysis, with intraclass correlation coefficients (ICC) and corresponding 95% confidence intervals calculated for each subscale. To assess the scale’s consistency over time, ICC was calculated using data collected from 53 randomly selected participants at a 2-week interval. Time consistency was evaluated using a two-way mixed-effects, absolute-agreement, single-measures ICC model (ICC (3,1)) with 95% confidence intervals (Koo and Li, 2016). Thresholds <0.50 were interpreted as poor, 0.50–0.74 as moderate, 0.75–0.89 as good, and ≥0.90 as excellent reliability. Convergent validity was assessed by computing Pearson correlation coefficients between the FORCES subscales and established measures of eating behavior and psychological constructs, including the TFEQ-R21, DTEDS, STAI-S, and STAI-T, as well as BMI. Statistical significance was determined at a threshold of *p* < 0.05.

## 3 Results

### 3.1 Sociodemographic characteristics

A total of 833 women participated in the study across two independent cross-sectional samples. The first sample was used for EFA (*n* = 416), whereas the second sample was used for CFA (*n* = 417). Table 1 presents the sociodemographic characteristics, anthropometric measurements, and FORCES score distributions of the participants. The mean age of the EFA group was 21.8 years (±6.1), and the mean age of the CFA group was 21.3 years (±5.3). The majority of participants in both groups were single (91.1% in the EFA group and 94.0% in the CFA group). The evaluation of anthropometric measurements found a mean BMI of 22.8 ± 3.8 kg/m^2^ in the EFA group and 22.4 ± 3.9 kg/m^2^ in the CFA group. The mean overall “*Rigid Control*” score for the EFA group was 28.4 ± 6.9, while for the CFA group, it was 28.8 ± 7.2. The mean overall “*Flexible Control*” score was determined to be 15.1 ± 3.3 in the EFA group and 14.6 ± 3.4 in the CFA group.

**Table 1:** Participants’ characteristics.

	EFA (*n* = 416)	CFA (*n* = 417)
Variables	*n* (%)	*n* (%)
Age (years)*	21.8 ± 6.1	21.3 ± 5.3
BMI (kg/m^2^)*	22.8 ± 3.8	22.4 ± 3.9
BMI group		
Underweight	40 (9.6)	47 (11.3)
Normal weight	286 (68.8)	293 (70.3)
Overweight	71 (17.1)	59 (14.1)
Obesity	19 (4.6)	18 (4.3)
Marital status		
Single	379 (91.1)	392 (94.0)
Married	37 (8.9)	25 (6.0)
Educational levels		
Primary/secondary school	3 (0.7)	3 (0.7)
High school	26 (6.3)	20 (4.8)
Associate’s degree/Bachelor’s degree	368 (88.5)	379 (90.9)
Postgraduate degree	19 (4.5)	15 (3.6)
FORCES total score*		
Rigid control*	28.4 ± 6.9	28.8 ± 7.2
Strict behaviors	10.6 ± 2.7	10.8 ± 2.7
Negative emotions	11.4 ± 3.7	11.6 ± 3.9
Worry	6.4 ± 2.1	6.5 ± 2.2
Flexible control*	15.1 ± 3.3	14.6 ± 3.4
Flexible beliefs	8.1 ± 1.9	7.9 ± 1.9
Positive emotions	7.0 ± 2.3	6.7 ± 2.2

*Mean ± SD, EFA: Exploratory Factor Analysis; CFA: Confirmatory Factor Analysis; BMI: Body Mass Index; FORCES: The Flexible or Rigid Control of Eating Scale.

### 3.2 Exploratory factor analysis

The factor structure of the Turkish version of the FORCES is presented in Table 2. Bartlett’s test of sphericity was significant (χ^2^ = 4474.8, *p* < 0.001), and the Kaiser–Meyer–Olkin (KMO) coefficient was 0.868, indicating that the dataset was suitable for factor analysis. EFA supported a five-factor structure consistent with the theoretical framework of the original FORCES and accounting for 62.85% of the total variance. The extracted factors corresponded to the predefined subdimensions of Strict Behaviors, Negative Emotions, Worry, Flexible Beliefs, and Positive Emotions. Eigenvalues ranged from 1.094 to 6.305, and factor loadings ranged from 0.589 (Item 16) to 0.921 (Item 18), indicating satisfactory item representation across all subdimensions.

**Table 2:** Factor structure of the Turkish version of the FORCES.

FORCES items	Factor				
	1	2	3	4	5
Eigenvalue (% explained variance)	1.918 (7.8)	6.305 (31.6)	1.094 (3.5)	1.619 (6.5)	2.988 (13.8)
Strict behaviors					
Item 1	0.655				
Item 2	0.762				
Item 3	0.604				
Item 4	0.604				
Item 5	0.705				
Negative emotions					
Item 6		0.812			
Item 7		0.818			
Item 8		0.909			
Item 9		0.855			
Item 10		0.620			
Worry					
Item 11			0.614		
Item 12			0.813		
Item 13			0.637		
Flexible beliefs					
Item 14				0.734	
Item 15				0.849	
Item 16				0.589	
Positive emotions					
Item 17					0.818
Item 18					0.921
Item 19					0.876

FORCES: The Flexible or Rigid Control of Eating Scale; Factor 1 (F1): Strict behaviors; Factor 2 (F2): Negative emotions; Factor 3 (F3): Worry; Factor 4 (F4): Flexible beliefs; Factor 5 (F5): Positive emotions.

### 3.3 Confirmatory factor analysis

The CFA results for the Turkish version of the FORCES are presented in Table 3. Prior to confirming the hypothesized five-factor first-order model, an alternative second-order model representing higher-order rigid and flexible control dimensions was examined in preliminary analyses. Although the second-order model demonstrated acceptable incremental fit indices (χ^2^/df = 3.708, SRMR = 0.067, RMSEA = 0.079, CFI = 0.993, IFI = 0.993, TLI = 0.991), the first-order model showed comparatively better overall fit (χ^2^/df = 2.655, CFI = 0.951, IFI = 0.951, SRMR = 0.035, RMSEA = 0.063), particularly with respect to SRMR and RMSEA values. The analysis also included examination of modification indices, which identified two theoretically justifiable covariances (e1–e4 and e9–e10) based on semantic proximity of the corresponding items. Collectively, these findings further support the conceptual distinction between rigid and flexible eating control dimensions and reinforce the structural validity of the Turkish FORCES (Figure 1).

**Table 3:** Model goodness-of-fit indices of the Turkish version of the FORCES.

Fit indices	Perfect fit	Acceptable fit	First-order CFA	Second-order CFA
χ^2^/df	0 ≤ χ^2^/df ≤ 3	3 ≤ χ^2^/df ≤ 4	2.655	3.708
SRMR	0 ≤ SRMR ≤ 0.05	0.05 ≤ SRMR ≤ 0.1	0.035	0.067
RMSEA	0 < RMSEA < 0.05	0.05 < RMSEA < 0.08	0.063	0.079
AGFI	0.95 ≤ AGFI ≤1	0.80 ≤ AGFI ≤0.90	0.886	0.837
GFI	0.95 ≤ GFI ≤ 1	0.80 ≤ GFI ≤ 0.90	0.916	0.991
CFI	0.95 ≤ CFI ≤ 1	0.90 ≤ CFI ≤ 0.95	0.951	0.993
IFI	0.95 ≤ IFI ≤ 1	0.90 ≤ IFI ≤ 0.95	0.951	0.993
TLI	0.95 ≤ TLI ≤ 1	0.90 ≤ TLI ≤ 0.95	0.940	0.991

FORCES: The Flexible or Rigid Control of Eating Scale; χ2/df: Chi-square/Degree of Freedom; SRMR: Standardized Root Mean Square Residual; RMSEA: Root Mean Square Error of Approximation; AGFI: Adjusted Goodness of Fit Index; GFI: Goodness of Fit Index; CFI: Comparative Fit Index; IFI: Incremental Fit Index; TLI: Tucker Lewis Index.

**Figure 1:**
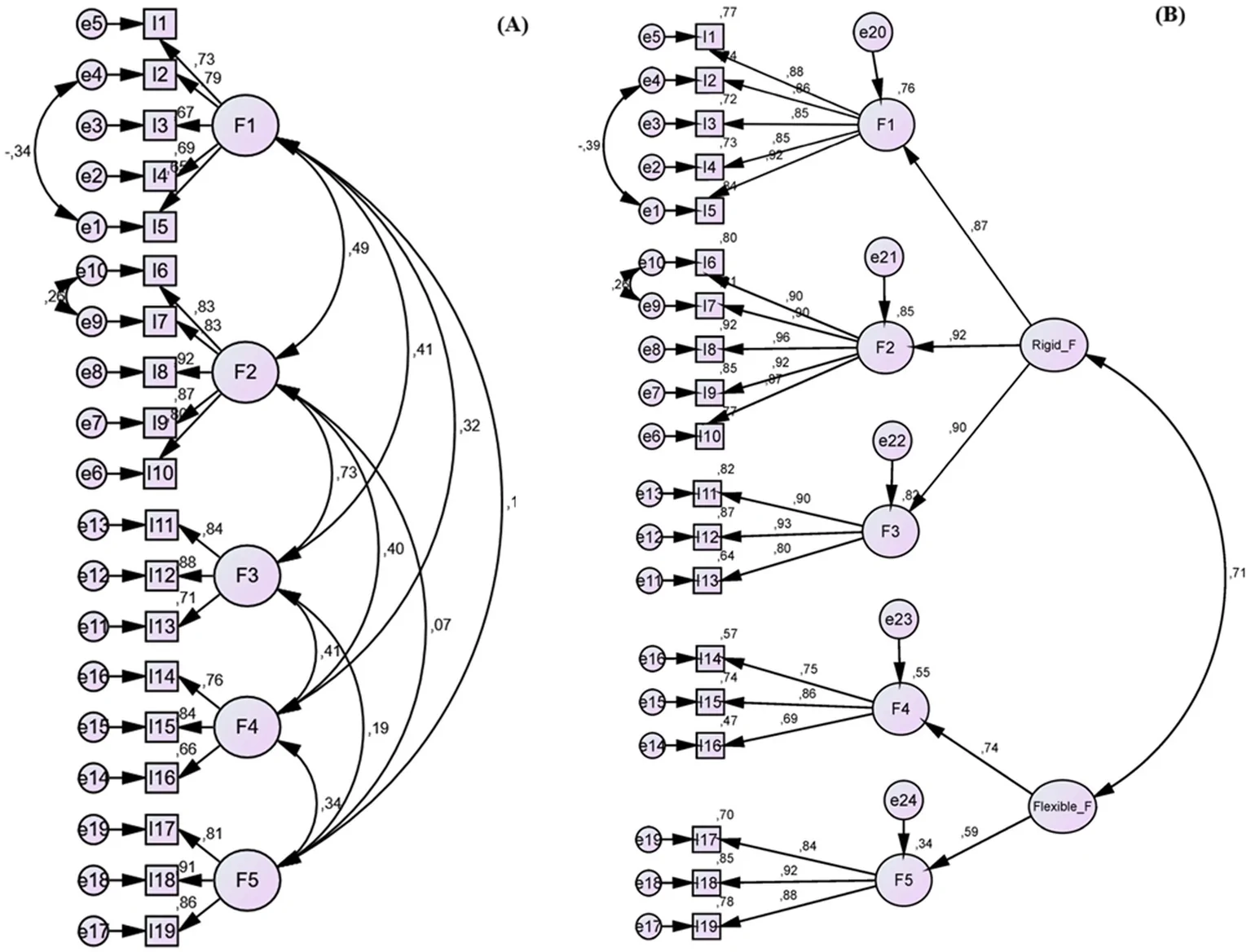
Path diagrams of the first-order **(A)** and second-order **(B)** confirmatory factor analyses for the Turkish version of the FORCES. **(A)** The first-order correlated five-factor model. **(B)** The second-order hierarchical model represents “Rigid Control” and “Flexible Control” as higher-order dimensions. F1: Strict behaviors, F2: Negative emotions, F3: Worry, F4: Flexible beliefs, F5: Positive emotions. To improve model fit, covariances were defined between error terms e1–e4 and e9–e10 according to modification indices. All standardized path coefficients presented are statistically significant (*p* < 0.001).

Item-level fit statistics from the Rasch model indicated satisfactory model fit between the model and the data across all 19 items. Both Infit and Outfit Mean Square (MnSq) values for all items fell within the acceptable range of 0.5 to 1.5 (Infit range: 0.811-1.418; Outfit range: 0.791-1.424), confirming that no items were misfitting or degrading the measurement model. Regarding measurement invariance, DIF analysis was conducted across BMI groups (participants classified as underweight or normal weight and participants classified as having overweight or obesity). The results revealed that of the 17 calculable items, 14 showed no significant DIF (adj. *p* > 0.05). Significant DIF was observed in only three items: Item 1 (*p* < 0.001) and Item 5 (*p* = 0.036) from the Rigid Control dimension, and Item 15 (*p* = 0.031) from the Flexible Control dimension (Supplementary Table S2). These findings support substantial measurement equivalence across BMI groups.

To evaluate the internal precision and local independence of the higher-order constructs, person reliability and MADaQ3 statistics were computed (Supplementary Table S3). Person reliability was excellent for rigid control (0.901) and very good for flexible control (0.807), indicating adequate discrimination across the latent trait continuum. The MADaQ3 values, assessing local item dependence, were 0.228 for rigid control and 0.370 for flexible control, indicating acceptable local dependence overall.

Furthermore, Person-Item maps (Wright maps) were generated to assess how well item difficulties align with participants’ trait levels (Supplementary Figure S1). Both the rigid control (Supplementary Figure S1A) and flexible control (Supplementary Figure S1B) maps demonstrated a satisfactory alignment between the distribution of participants’ abilities (upper panel histograms) and the item thresholds (lower panel). The items adequately covered the range of the latent traits without clustering exclusively at the extremes, indicating good targeting.

### 3.4 Reliability analysis of FORCES

The reliability analyses of the Turkish version of the FORCES are presented in Table 4. Internal consistency coefficients demonstrated satisfactory to excellent reliability across all five subdimensions. Cronbach’s alpha values ranged from 0.766 to 0.917, while McDonald’s omega coefficients ranged from 0.775 to 0.918, supporting the internal consistency of the multidimensional structure. Item–total correlation coefficients varied between 0.514 and 0.846, indicating acceptable to strong item discrimination across the scale. In addition, the “alpha if item deleted” values suggested that removal of any individual item did not meaningfully improve subscale reliability. The overall scale demonstrated high internal consistency (α = 0.874; ω = 0.880), while subscale reliability coefficients ranged from 0.766 to 0.917 for Cronbach’s alpha and from 0.775 to 0.918 for McDonald’s omega. Collectively, these findings support the reliability and internal coherence of the Turkish version of the FORCES. Additional psychometric analyses provided further evidence regarding the convergent validity and internal reliability of the FORCES subdimensions (Supplementary Table S4). Average Variance Extracted (AVE) values ranged from 0.502 to 0.743, exceeding the recommended threshold of 0.50 across all factors. Similarly, Composite Reliability (CR) coefficients ranged from 0.798 to 0.929, indicating satisfactory to excellent construct reliability. Overall, these findings provide additional support for the convergent validity and structural validity of the Turkish FORCES.

**Table 4:** Internal reliability of the Turkish version of the FORCES.

Items	α coefficient	McDonald’s ω	Mean	SD	Item-total correlation	α if item deleted
Item 1	0.814	0.817	2.1	0.6	0.588	0.783
Item 2			2.4	0.7	0.649	0.764
Item 3			2.0	0.6	0.612	0.776
Item 4			2.4	0.8	0.579	0.789
Item 5			1.8	0.6	0.600	0.780
Item 6	0.917	0.918	2.3	0.8	0.768	0.902
Item 7			2.3	0.8	0.803	0.895
Item 8			2.3	0.8	0.844	0.886
Item 9			2.4	0.8	0.813	0.893
Item 10			2.2	0.8	0.704	0.914
Item 11	0.810	0.822	2.2	0.8	0.667	0.732
Item 12			2.2	0.8	0.752	0.643
Item 13			2.1	0.8	0.594	0.805
Item 14	0.766	0.775	2.7	0.7	0.623	0.659
Item 15			2.7	0.7	0.666	0.611
Item 16			2.7	0.8	0.548	0.758
Item 17	0.905	0.906	2.4	0.8	0.776	0.893
Item 18			2.4	0.8	0.846	0.834
Item 19			2.3	0.8	0.811	0.864
FORCES total	0.874	0.880	2.3	0.4	–	–

FORCES: The Flexible or Rigid Control of Eating Scale; SD: Standard deviation.

The test–retest analysis results’ Intraclass Correlation Coefficient (ICC) values and 95% confidence ranges are shown in Table 5. The test–retest reliability coefficients for the subdimensions of the scale ranged between 0.726 and 0.821. The highest temporal stability was found in the “*Worry*” (ICC = 0.821) and “*Strict Behaviors*” (ICC = 0.817) subdimensions (*p* < 0.01).

**Table 5:** Test–retest reliability (ICC).

FORCES	ICC	95% Confidence Interval		*p*
		Lower	Upper	
Rigid control	0.786	0.630	0.877	<0.001
Strict behaviors	0.817	0.683	0.894	<0.001
Negative emotions	0.746	0.560	0.853	<0.001
Worry	0.821	0.690	0.897	<0.001
Flexible control	0.732	0.536	0.845	<0.001
Flexible beliefs	0.726	0.525	0.842	<0.001
Positive emotions	0.741	0.551	0.850	<0.001

FORCES: The Flexible or Rigid Control of Eating Scale; Factor 1: Strict behaviors; Factor 2: Negative emotions; Factor 3: Worry; Factor 4: Flexible beliefs; Factor 5: Positive emotions; ICC: Intraclass Correlation Coefficient. Test–retest reliability was calculated in a subsample using a two-way mixed-effects, absolute-agreement, single-measures model [ICC(3,1)] over a 2-week interval (*n* = 53).

### 3.5 Convergent and criterion-related validity

Table 6 presents the bivariate correlations between FORCES subscales and external validity criteria across the EFA and CFA samples, while Figure 2 provides a complementary heatmap visualization of these associations (Figure 2A: EFA; Figure 2B: CFA). As shown in both the tabular and graphical representations, the “*Worry”* subscale demonstrated a statistically significant positive association with TFEQ-Emotional Eating, with correlation coefficients of *r* = 0.460 in the EFA sample and *r* = 0.407 in the CFA sample (*p* < 0.01). Similarly, the “*Negative Emotions”* subscale was moderately and consistently correlated with the “DTEDS-11 Eating” dimension in both samples (EFA: *r* = 0.422; CFA: *r* = 0.493), indicating convergent validity with maladaptive cognitive eating patterns. Significant associations were also observed between “*Strict Behaviors*” and “TFEQ–Cognitive Restraint” (EFA: *r* = 0.150; CFA: *r* = 0.178), whereas the higher-order “*Rigid Control*” construct showed stable positive correlations with “TFEQ–Emotional Eating” across both independent samples (EFA: *r* = 0.379; CFA: *r* = 0.373). With respect to anthropometric variables, higher BMI values were associated with increased levels of anxiety-related constructs, including “*Worry*” (EFA: *r* = 0.347; CFA: *r* = 0.255) and “*Negative Emotions*” (EFA: *r* = 0.226; CFA: *r* = 0.203). Age exhibited weak but statistically significant positive correlations with “*Rigid Control*” in both samples (EFA: *r* = 0.147; CFA: *r* = 0.178). Overall, the concordant correlation patterns observed in Table 6 and visually reinforced in Figure 2 support the construct validity of the FORCES, demonstrating consistent associations with eating pathology–related, emotional, and cognitive measures across independent samples.

**Table 6:** Correlation analysis between FORCES subscales and age, BMI, DTEDS-11, TFEQ, and STAI subscales for EFA and CFA.

	Strict behaviors	Negative emotions	Worry	Rigid control	Flexible beliefs	Positive emotions	Flexible control
EFA (*n* = 416)							
Strict behaviors	–						
Negative emotions	0.459**	–					
Worry	0.351**	0.641**	–				
Rigid control	0.738**	0.902**	0.778**	–			
Flexible beliefs	0.193**	0.290**	0.249**	0.304**	–		
Positive emotions	0.045	0.008	0.166**	0.072	0.283**	–	
Flexible control	0.141**	0.172**	0.255**	0.223**	0.764**	0.835**	–
DTEDS-11-Eating	0.200**	0.422**	0.367**	0.412**	0.141**	−0.029	0.062
DTEDS-11-General	0.042	0.247**	0.255**	0.224**	0.056	0.043	0.061
DTEDS-11-Total	0.137**	0.373**	0.347**	0.356**	0.111**	0.006	0.068
TFEQ-Uncontrolled eating	0.107**	0.281**	0.449**	0.326**	0.133**	0.171**	0.191**
TFEQ-Cognitive restraint of eating	0.150**	0.311**	0.421**	0.350**	0.128**	0.128**	0.160**
TFEQ-Emotional eating	0.179**	0.322**	0.460**	0.379**	0.188**	0.097*	0.173**
STAI-State	−0.011	0.045	0.121**	0.056	0.001	0.113**	0.077
STAI- Trait	−0.181**	0.077	0.086	−0.004	0.163**	0.027	0.112**
Age	0.134**	0.089	0.158**	0.147**	0.008	−0.013	−0.004
BMI	0.084	0.226**	0.347**	0.257**	0.017	−0.016	−0.032
CFA (*n* = 417)							
Strict behaviors	–						
Negative emotions	0.469**	–					
Worry	0.365**	0.662**	–				
Rigid control	0.734**	0.909**	0.793**	–			
Flexible beliefs	0.261**	0.324**	0.319**	0.367**	–		
Positive emotions	0.093	0.066	0.189**	0.127**	0.340**	–	
Flexible control	0.210**	0.228**	0.305**	0.293**	0.793**	0.843**	–
DTEDS-11-Eating	0.242**	0.493**	0.369**	0.465**	0.169**	0.001	0.097*
DTEDS-11-General	0.118*	0.316**	0.303**	0.305**	0.122*	0.140*	0.161**
DTEDS-11-Total	0.199**	0.444**	0.367**	0.422**	0.160*	0.073	0.138*
TFEQ-Uncontrolled eating	0.149*	0.303**	0.435**	0.349**	0.119*	0.167**	0.177**
TFEQ-Cognitive restraint of eating	0.178**	0.318**	0.373**	0.349**	0.153**	0.145*	0.182**
TFEQ- Emotional eating	0.196**	0.331**	0.407**	0.373**	0.159**	0.102*	0.157*
STAI-State	0.013	0.060	0.082	0.062	−0.007	0.171**	0.107*
STAI-Trait	−0.049	0.071	0.123*	0.057	0.194**	0.096	0.174**
Age	0.132*	0.149*	0.163**	0.178**	0.001	−0.101*	−0.065
BMI	−0.018	0.203**	0.255**	0.179**	−0.013	−0.091	−0.067

FORCES: The Flexible or Rigid Control of Eating Scale; BMI: Body Mass Index; DTEDS-11: The Dichotomous Thinking in Eating Disorders Scale; TFEQ: Three-Factor Eating Questionnaire; STAI: State Trait Anxiety Inventory. *p < 0.05, **p < 0.01.

**Figure 2:**
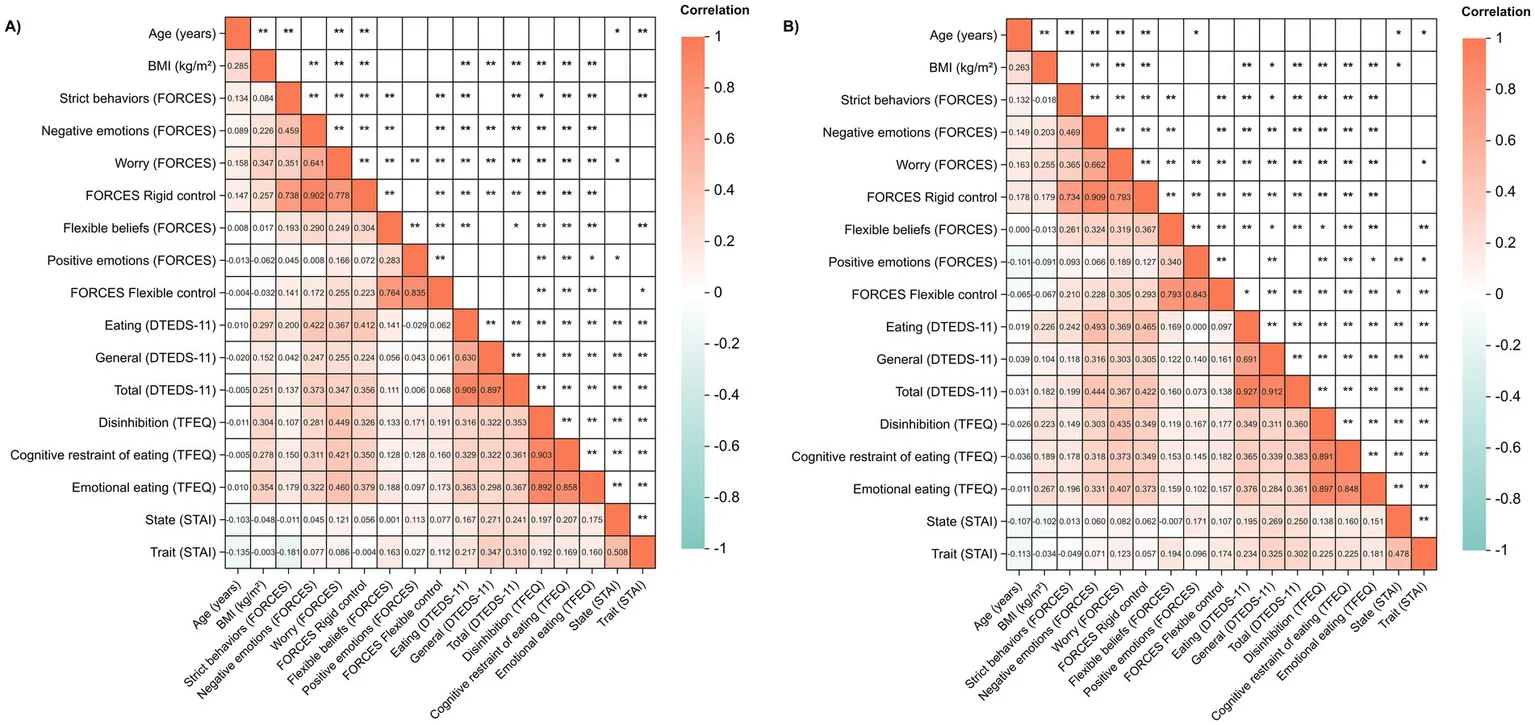
Heatmap of bivariate correlations between FORCES subscales and related psychological and behavioral measures across the EFA **(A)** and CFA **(B)** samples.

## 4 Discussion

In this study, the Turkish adaptation of FORCES was conducted, and the psychometric properties of the scale were evaluated in a large sample of women. The aim was to obtain a tool that can measure flexible and rigid eating control dimensions in a valid and reliable manner within the Turkish cultural context, thereby contributing to a more precise and multidimensional assessment of eating behavior. The results of the study indicate that the original factor structure, consisting of 19 items and five subscales (“Strict Behaviors,” “Negative Emotions,” “Worry,” “Flexible Beliefs,” and “Positive Emotions”), was preserved in the Turkish form, and that the scale generally exhibits good psychometric properties in terms of both construct validity and reliability indicators.

### 4.1 Assessment of construct validity and factor structure

The replication of the factor structure across two independent samples using EFA and CFA offers compelling evidence for the construct validity of the Turkish version of FORCES. The EFA conducted on the first subsample (*n* = 416) determined that the five-factor structure was the most appropriate model, considering the eigenvalues, total variance explained, and factor loadings. Bartlett’s sphericity test (χ^2^ = 4474.8, *p* < 0.001) and the KMO coefficient of 0.868 indicate that the dataset is suitable for factor analysis. This analysis revealed that all item factor loadings ranged between 0.589 and 0.921, and the five-factor solution explained 62.85% of the total variance. Furthermore, no significant cross-loadings were found between factors, and no items needed to be removed from the scale.

The CFA conducted on the second subsample confirmed the five-factor structure obtained with EFA. The obtained fit indices (χ^2^/df = 2.655; CFI = 0.951; TLI = 0.940; RMSEA = 0.063; SRMR = 0.035; GFI = 0.916; AGFI = 0.886) indicate that the model showed a generally good and acceptable fit. To improve model fit, two covariances (e1-e4 and e9-e10) were defined among the error terms, considering theoretical and semantic proximities. After these changes, all path coefficients retained their significance (*p* < 0.001). These results show that the five-factor structure reported by Nicholls et al. (2025) in samples of women can also be reproduced in young adult women from Türkiye (Nicholls et al., 2025). Similarly, Péneau et al. (2022) reported that the two-dimensional (flexible/rigid) structure was validated with good fit indices in their validity-reliability study of the Flexible and Rigid Cognitive Restriction Scale in France (Péneau et al., 2022). The emergence of flexible and rigid control dimensions as separable structures across different cultures supports the findings of our study. Taken together, these results indicate that the Turkish version of FORCES is a measurement tool with construct validity that represents both flexible and rigid eating control dimensions in line with theoretical expectations. The fact that the scale’s subscales can be conceptually grouped under “*Rigid Control*” (“Strict Behaviors,” “Negative Emotions,” “Worry”) and “*Flexible Control*” (“Flexible Beliefs,” “Positive Emotions”) clusters, as in the original study, appears to largely achieve the goal of distinguishing flexible and rigid control.

The application of IRT provided deep insights into the psychometric robustness of the Turkish version of FORCES. The ideal Infit and Outfit statistics across all items suggest that the scale is free from noisy or misfitting items, ensuring high measurement precision.

An important finding of the current study is the providing preliminary evidence of measurement equivalence across BMI groups. The DIF analysis confirmed that more than 80% of the calculable items function equivalently regardless of BMI classification status. Although minor DIF was detected in three items (Items 1, 5, and 15), such negligible variance in a 19-item instrument does not threaten the overall construct validity. Consequently, the FORCES can be considered a generally unbiased and reliable instrument for comparative clinical and behavioral research targeting obesity, eating disorders, and diverse BMI groups.

The application of Rasch analysis provided robust evidence of the FORCES’ precision and appropriateness in this population. The high person reliability indices (>0.80) for both higher-order dimensions ensure that the scale can consistently and accurately differentiate individuals based on their eating control styles. Although the MADaQ3 value for the flexible control dimension was relatively elevated (0.370), values of this magnitude may occur in shorter conceptually related subscales and did not indicate substantial local dependence concerns.

Moreover, the Person-Item (Wright) maps (Supplementary Figure S1) offer additional evidence regarding the alignment between item difficulty and participant ability levels of the scale’s targeting capability. The successful alignment between participant trait levels and item difficulty thresholds confirms that the scale items are neither too easy nor too difficult for the target population. This optimal targeting indicates the absence of severe ceiling or floor effects, suggesting that both the rigid and flexible dimensions successfully capture the full spectrum of eating regulation behaviors among young adult women.

### 4.2 Evaluation of reliability findings

The internal consistency coefficients obtained in the study indicate that the Turkish version of FORCES is also adequate in terms of reliability. In the EFA sample (*n* = 416), the Cronbach’s alpha coefficients for the subscales were found to be 0.814 for Strict Behaviors, 0.917 for Negative Emotions, 0.810 for Worry, 0.766 for Flexible Beliefs, and 0.905 for Positive Emotions. These values are above the 0.70 threshold accepted in psychometric measurement literature and indicate a “good” or “very good” level of internal consistency for most subscales.

Similarly, the McDonald *ω* coefficients ranging from 0.775 to 0.918 and the ICC values for test–retest reliability ranging from 0.726 to 0.821 across all subscales indicate that the scale has a high level of reliability in terms of both internal consistency and stability over time. When compared to the internal consistency coefficients reported by Nicholls et al. (2025) (α = 0.82–0.94 range), the coefficients obtained in the Turkish form are in a similar range, suggesting that the scale can produce reliable results consistently across different cultural contexts (Nicholls et al., 2025). Similarly, Péneau et al. (2022) reported McDonald’s ω coefficients for the flexible and rigid restraint dimensions in the range of 0.74–0.77 and interpreted these values as indicative of acceptable levels of internal consistency (Péneau et al., 2022). Taken together, these reliability findings indicate that the Turkish FORCES not only demonstrates strong internal consistency but also yields stable scores over time. This suggests that the scale can reliably capture both cognitive and emotional components of flexible and rigid eating control across repeated assessments, supporting its suitability for both cross-sectional and longitudinal research designs. Beyond simple replication, the present study contributes to the multidimensional assessment of eating regulation by evaluating the structural validity of rigid and flexible control within a Turkish sociocultural context.

### 4.3 Relationships with related constructs and theoretical consistency

This study found that the FORCES subscales showed significant relationships with other psychological constructs related to eating behavior (cognitive restraint, uncontrolled eating, emotional eating, and dichotomous thinking related to eating). The Strict Behaviors, Negative Emotions, and Worry subscales showed weak to moderate positive correlations with both uncontrolled eating, cognitive restraint, and emotional eating (TFEQ-R21) and eating-related dichotomous thinking (DTEDS-11) scores (*r* = 0.10-0.49, *p* < 0.01). “Flexible Beliefs” and “Positive Emotions,” along with the “*Flexible Control*” total score, also showed significant positive correlations with these variables, albeit at a lower level (*r* = 0.10–0.25, mostly *p* < 0.01). Importantly, the observed positive associations involving “*Flexible Control*” should not be interpreted as evidence of maladaptive regulation. Rather, these findings may reflect a cognitively engaged and goal-oriented approach to eating regulation within sociocultural environments where dietary monitoring and body weight awareness are common. Flexible control, therefore, should not be conceptualized as the absence of control, but as a qualitatively different regulatory style that allows adaptability while maintaining engagement with dietary goals.

The literature indicates that rigid eating control is associated with higher psychological distress, eating disorder symptoms, body dissatisfaction, and a more negative body image. In contrast, flexible control has been reported to be associated with longer-term weight management and relatively more positive psychological outcomes (Conlin et al., 2021; Linardon et al., 2021; Péneau et al., 2022). Moreover, a recent systematic review (Mason et al., 2024) indicates that the relationship between dietary restriction and binge eating is sensitive to mediating/moderating variables, particularly negative affect and cognitive flexibility. The consistency of the correlation pattern obtained in our study with this literature demonstrates that the Turkish version of FORCES is also supported in terms of convergent and discriminant validity.

In particular, the Worry subscale was found to be moderately positively related to emotional eating in both subsamples (EFA: *r* = 0.460; CFA: *r* = 0.407) and the Negative Emotions subscale being significantly related to the “Eating” subscale of the eating-related dichotomous thinking scale (EFA: *r* = 0.422; CFA: *r* = 0.493) supports that the Rigid Control dimensions exhibit expected relationships with structures linked to eating psychopathology. Additionally, weak but significant positive relationships between age and Rigid Control scores (*r* = 0.15–0.18) and the increase in Worry and Negative Emotions scores as BMI increases (*r* = 0.26–0.35 for Worry; Negative Emotions *r* = 0.20–0.23) indicate that the scale is sensitive to weight-related emotional and cognitive processes.

Notably, the absence of significant associations between BMI and certain FORCES subdimensions, particularly those reflecting flexible control, suggests that not all forms of eating regulation are directly linked to body weight. In contrast to the observed positive associations between BMI and rigid control components such as Worry and Negative Emotions, flexible control appears to represent a qualitatively different regulatory process.

The differing magnitudes of associations observed between rigid and flexible control dimensions and related psychological constructs further support the multidimensional nature of eating control. While rigid control, characterized by heightened worry, negative affect, and strict rule adherence, may contribute to maladaptive eating patterns and weight-related outcomes, flexible control may function as a more adaptive regulatory style that emphasizes behavioral flexibility, moderation, and contextual decision-making rather than caloric restriction per se. Consequently, flexible control may be more closely associated with psychological well-being and sustainable eating behaviors than with direct variations in BMI. This interpretation is consistent with a growing body of literature suggesting that adaptive eating regulation does not necessarily translate into lower body weight but instead reflects healthier cognitive and behavioral relationships with food.

Numerous studies indicate that intuitive and mindful eating are positively associated with both body weight regulation and psychological well-being (Akırmak et al., 2018; Ayyıldız et al., 2022; Çelik and Bayır, 2023; Dinç et al., 2025; Giacone et al., 2024; Hamurcu et al., 2023; Önal et al., 2023). These findings consistently demonstrate associations between adaptive eating styles and BMI, depression and anxiety symptoms, perceived stress, and healthier dietary behaviors. In particular, greater engagement in mindful and intuitive eating has been linked to more favorable psychological and behavioral outcomes among Turkish adults and university students. Within this framework, adaptive regulatory approaches, such as flexible eating control, may function as protective factors, particularly among young adult women, by promoting balanced eating patterns and reducing maladaptive cognitive-emotional responses to food. Accordingly, the flexible control subdimensions of the FORCES may offer a valuable framework for investigating adaptive eating processes in conjunction with these constructs.

Building on this framework, the differential associations observed between FORCES subscales and state versus trait anxiety provide further insight into the psychological mechanisms underlying eating control. State anxiety, reflecting transient emotional responses to situational stressors, appears to be more closely linked to immediate fluctuations in eating-related worry and emotional reactivity. In contrast, trait anxiety represents a more stable predisposition toward anxiety, which may manifest in enduring cognitive patterns such as rigid rule adherence and heightened sensitivity to perceived dietary violations. This distinction suggests that Rigid Control dimensions may be influenced by both situational emotional states and stable cognitive-affective dispositions. Collectively, these findings highlight the multidimensional interplay between anxiety and eating regulation, indicating that both momentary emotional states and enduring personality traits contribute to the expression of maladaptive eating control patterns.

### 4.4 Cultural context and contributions specific to the Turkish sample

The sample of this study consists of young adult women living in Türkiye. Young women are a group for whom flexible and rigid eating control is particularly critical, due to both the intensity of societal pressures regarding body image and the prevalence of dieting behaviors. Indeed, although the average BMI in our sample was within the normal range (22.8 and 22.4 kg/m^2^), the increase in Worry and Negative Emotions scores accompanying the increase in BMI indicates that the emotional burden associated with weight may be more intense, especially among young women with BMI values within or above the normal range. Current studies show that eating behaviors are shaped by many genetic, environmental, cultural, and media-based factors, and that religious-cultural norms and social body ideals can also be decisive in eating styles (Biçer and Kahrıman, 2025; Koç, 2025; Meriç and Ayhan, 2025). In addition, it has been reported that family meal routines, traditional eating practices, and intergenerational transmission of eating habits shape eating styles in the long term, particularly during childhood and young adulthood (Koç, 2025). This developmental specificity should be considered when interpreting the prominence of Rigid Control dimensions, as these patterns may be particularly accentuated during early adulthood due to heightened psychosocial vulnerability and identity formation processes.

Beyond structural validation, it is important to consider that the phenomenological experience and social meaning of eating control may vary across cultural contexts. In Türkiye, eating behaviors are shaped by a complex interplay between traditional dietary practices, characterized by structured meals, strong family involvement, and cultural norms surrounding food sharing, and rapidly evolving modern influences, including increased exposure to globalized diet culture, social media, and Westernized body ideals. Within this hybrid sociocultural environment, rigid control strategies may be reinforced not only through individual dieting efforts but also through culturally embedded norms emphasizing discipline, self-regulation, and moral evaluations of food choices.

Importantly, the distinction between rigid and flexible control may not be uniformly interpreted across cultural contexts. While flexible control is typically conceptualized as adaptive in Western literature, in certain sociocultural settings it may be perceived as a lack of discipline or insufficient adherence to dietary norms. Conversely, rigid control, despite its association with maladaptive psychological outcomes, may be socially reinforced or even valorized due to its alignment with culturally endorsed ideals of self-control and dietary restraint. This highlights the need to interpret the FORCES dimensions not only as psychological constructs but also as culturally situated behavioral patterns shaped by social expectations and normative beliefs.

In Türkiye, socio-cultural messages that include both pressures to be thin and rigid adherence to certain dietary patterns (e.g., specific “healthy” categories) may reinforce strict control strategies among young women. In this context, the ability of the Turkish FORCES to separately assess the emotional (guilt, disappointment, anxiety) and cognitive (dichotomous rules, “completely abandoning a broken diet,” etc.) dimensions associated with strict control is an important advantage (Horovitz and Argyrides, 2023; Papini et al., 2022). Furthermore, the fact that the study was conducted on women provides unique data on how flexible and rigid eating control is structured during young adulthood. The lack of a valid measurement tool for assessing flexible and rigid eating control in the Turkish sociocultural context creates a significant gap in both the academic literature and nutrition counseling practice. The validity and reliability of this scale’s Turkish adaptation will enable the early detection of unhealthy control patterns in young women and the promotion of more flexible, sustainable nutrition strategies in future intervention and follow-up studies. Accordingly, the differentiation between rigid and flexible control provided by the FORCES may capture culturally nuanced variations in how individuals internalize and enact eating-related norms within the Turkish sociocultural framework.

### 4.5 Implications for research and practice

The findings of the present study suggest that the Turkish version of the FORCES may serve as a clinically and methodologically useful instrument for assessing eating control styles in both research and applied settings. The scale may assist dietitians, psychologists, and mental health professionals in identifying whether individuals predominantly rely on more rigid or more flexible forms of eating regulation, thereby facilitating more individualized intervention planning. In particular, elevated scores on Rigid Control dimensions, particularly Worry and Negative Emotions, together with higher BMI, may indicate the need for cognitive-behavioral approaches targeting guilt-related cognitions, anxiety, emotional dysregulation, and maladaptive weight-control beliefs. Conversely, individuals characterized by low flexible control and high rigid control may particularly benefit from interventions emphasizing intuitive eating, mindful eating, self-compassion, and cognitive flexibility (Conlin et al., 2021; Linardon et al., 2021).

From a research perspective, the Turkish FORCES may contribute to a broader understanding of eating-related psychopathology by enabling the multidimensional assessment of eating control processes. The scale may be examined in relation to a wide range of constructs, including eating disorder symptoms, orthorexia nervosa tendencies, body dissatisfaction, intuitive eating, mindful eating, and broader psychological symptom profiles.

Recent evidence suggests that orthorexic tendencies are closely associated with perfectionism and rigid dietary rules (Horovitz and Argyrides, 2023). Studies conducted in Türkiye have further highlighted significant associations between eating-related problems, appearance-oriented social media exposure, and problematic social media use, particularly among adolescents and young adults (Hamurcu et al., 2023; Meriç et al., 2024; Üstündağ et al., 2024; Yılmaz et al., 2025). These findings underscore the potential value of examining flexible and rigid eating control as distinct regulatory processes that may contribute to the development and maintenance of maladaptive eating behaviors.

Overall, the present findings demonstrate that the Turkish version of the FORCES retains the original five-factor structure and exhibits satisfactory psychometric properties in a sample of young adult women. The distinction between flexible and rigid eating control appears to be particularly relevant for understanding maladaptive eating patterns and identifying individuals who may be vulnerable to eating-related psychopathology.

Future studies involving male participants, gender-diverse samples, clinical populations, and culturally diverse subgroups are needed to further establish the generalizability of the Turkish FORCES. Longitudinal studies examining the predictive role of rigid and flexible eating control in weight trajectories, binge eating, dieting cycles, and psychological well-being may further clarify the long-term behavioral and psychological consequences of distinct eating regulation styles (Conlin et al., 2021; Mason et al., 2024). In addition, examining the sensitivity of FORCES subdimensions to change during intuitive and mindful eating-based interventions may help clarify the psychological mechanisms underlying adaptive eating regulation.

### 4.6 Limitations

Several limitations should be considered when interpreting the findings of the present study. First, the sample consisted exclusively of women and was largely composed of young, educated university students, limiting the generalizability of the findings to broader, more demographically diverse populations. Although this sampling approach allowed for methodological comparability with the original FORCES validation studies and enabled a more focused evaluation within a relatively homogeneous group, eating control is not a construct unique to women or young adults. Accordingly, the psychometric properties observed in the present study may not fully generalize to men, older individuals, or populations with different sociocultural and educational backgrounds.

The relatively narrow age distribution of the sample should also be considered. Eating control is a developmentally sensitive construct that may vary across the lifespan as a function of emotional regulation capacity, life experience, metabolic changes, and exposure to sociocultural pressures. Young adulthood, in particular, is often characterized by heightened body image concerns and increased engagement with dieting behaviors, factors that may intensify both rigid and flexible forms of eating control. In contrast, eating behaviors in older populations may be shaped more strongly by long-standing habits and less by appearance-related pressures. Therefore, caution is warranted when generalizing the present findings beyond young adult women.

Another important limitation is the absence of formal psychiatric and eating disorder screening procedures. Psychological conditions such as depression and anxiety may influence eating behavior, cognitive control processes, and emotional regulation, potentially affecting eating control patterns. Similarly, the inclusion of individuals with subclinical eating pathology cannot be ruled out. Future studies incorporating structured clinical assessments and more heterogeneous samples would provide a more comprehensive understanding of these relationships.

In addition, measurement invariance across relevant demographic and clinical subgroups was not evaluated. Although the five-factor structure was supported through EFA and CFA, the absence of invariance testing limits conclusions regarding the structural equivalence of the scale across populations differing in age, educational background, or clinical characteristics. Future studies employing multi-group CFA are needed to evaluate configural, metric, and scalar invariance and to establish the broader applicability of the Turkish FORCES across diverse populations.

The cross-sectional design of the study also precludes causal interpretations. Although significant associations were identified between eating control dimensions and various psychological and anthropometric variables, the temporal direction and potential reciprocal nature of these relationships remain unclear. Longitudinal studies are therefore needed to clarify the developmental and predictive significance of rigid and flexible eating control.

Furthermore, all data were obtained using self-report measures, which may be affected by social desirability bias, recall inaccuracies, and response distortion. In particular, height and weight were self-reported, which may have introduced additional reporting bias in BMI calculations. Although validated assessment tools were employed, these limitations should be considered when interpreting the findings.

Finally, regional and cultural heterogeneity within Türkiye may also influence eating-related attitudes and behaviors. Dietary practices, body image ideals, and sociocultural meanings attributed to eating may differ substantially across regions and communities. Consequently, the interpretation of eating control constructs should be considered within their broader sociocultural context. Future studies may benefit from employing broader psychometric approaches, including invariance testing and alternative factorial models, to further evaluate the structural stability of the Turkish FORCES across diverse populations.

## 5 Conclusion

The present study demonstrated that the Turkish adaptation of the FORCES is a reliable and psychometrically sound instrument for assessing multidimensional eating control among women in Türkiye. The findings supported the original five-factor structure of the scale and confirmed the distinction between rigid and flexible forms of eating regulation within a Turkish sociocultural context. The observed associations with eating behaviors, cognitive rigidity, emotional eating, anxiety, and BMI further support the construct validity of the Turkish FORCES and are consistent with existing theoretical and empirical literature.

The findings further support the conceptual distinction between rigid and flexible eating control as related yet distinct regulatory processes. Whereas rigid control was more strongly associated with maladaptive emotional and cognitive patterns, flexible control appeared to reflect a comparatively more adaptive regulatory style. In this respect, the FORCES provides a more nuanced assessment of eating control than traditional unidimensional restraint-based measures by simultaneously capturing emotional, cognitive, and behavioral aspects of dietary regulation.

By providing the first culturally adapted Turkish instrument capable of differentiating flexible and rigid eating control, the present study addresses an important gap in the literature and contributes to the growing cross-cultural investigation of eating regulation. The scale may facilitate future research examining eating-related psychopathology, body image concerns, intuitive and mindful eating, and sociocultural influences on dietary behavior. In addition, the Turkish FORCES may serve as a useful tool in preventive screening, clinical assessment, and intervention planning, particularly among young adult women exposed to dieting pressures and appearance-related sociocultural influences.

Overall, the findings support the applicability of the FORCES framework within the Turkish context and highlight the importance of multidimensional approaches to eating regulation. Future longitudinal and cross-cultural studies may further clarify how rigid and flexible eating control contribute to psychological well-being, maladaptive eating patterns, and long-term health outcomes across diverse populations.

## Data Availability

Data will be made available on request by the corresponding author.
