# Supplementary material for: Psychometric properties of the Turkish version of the Flexible or Rigid Control of Eating Scale in women
Source: Front Psychol. 2026 Sep 17;17:1826677. doi: 10.3389/fpsyg.2026.1826677 (PMC13627051; doi:10.3389/fpsyg.2026.1826677)
Supplement: Supplementary file 1 [file Image_1.pdf]

Supplementary Figure 1. Person-Item maps (Wright maps) for the Rigid Control (A) and Flexible Control (B) higher-order dimensions.

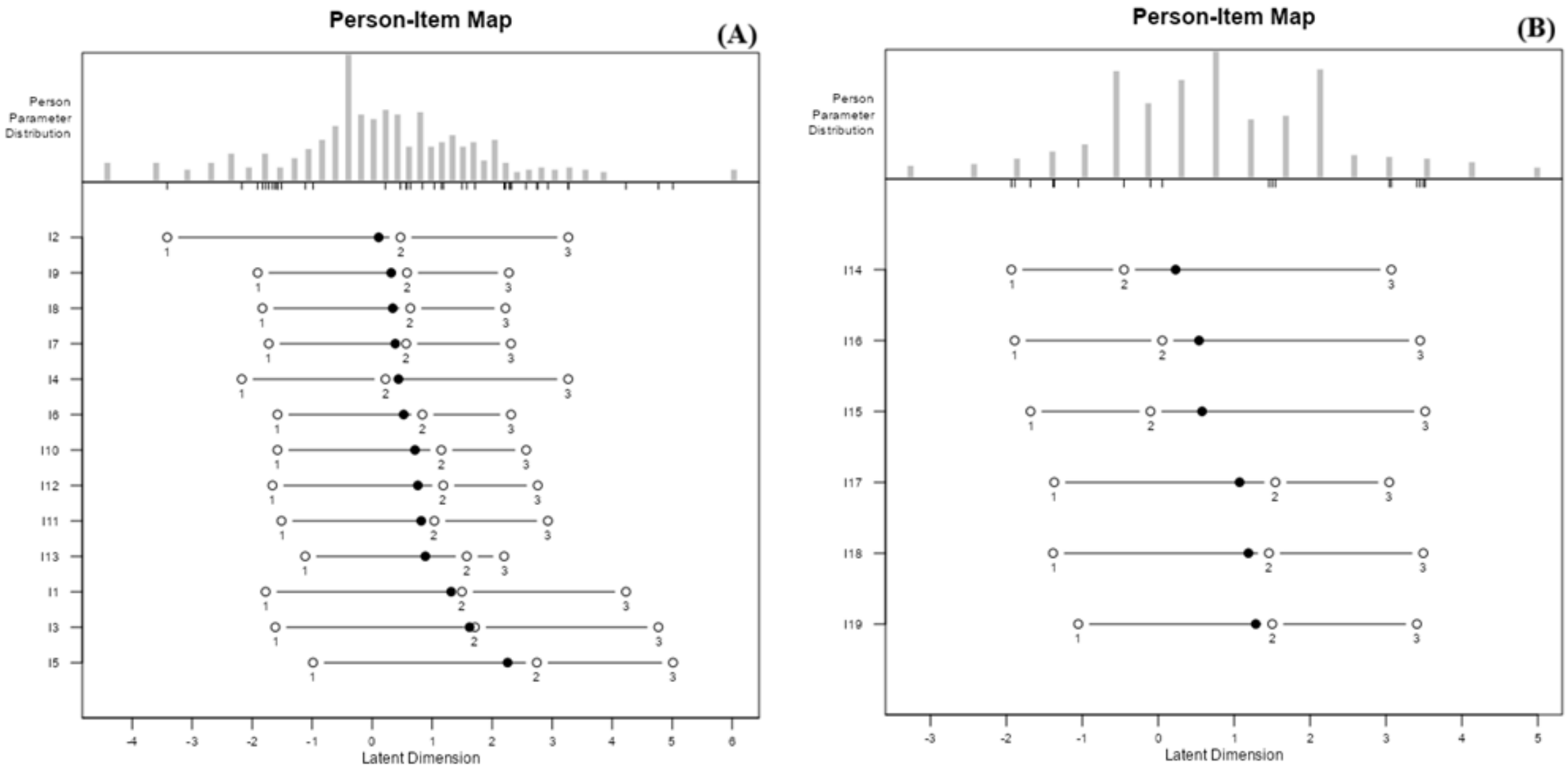

Note: The upper panel of each map shows the distribution of person measures (participant trait levels). The lower panel displays the item difficulty parameters and thresholds along the same latent logit scale. Map (A) represents the 13 items of the Rigid Control dimension, and Map (B) represents the 6 items of the Flexible Control dimension. The alignment between person distributions and item thresholds indicates adequate targeting of the measurement items for both constructs.
