# Supplementary material for: Psychometric properties of the Turkish version of the Flexible or Rigid Control of Eating Scale in women
Source: Front Psychol. 2026 Sep 17;17:1826677. doi: 10.3389/fpsyg.2026.1826677 (PMC13627051; doi:10.3389/fpsyg.2026.1826677)
Supplement: Supplementary file 2 [file Table_1.pdf]

Supplementary Table S1. Final Turkish Version of the Flexible or Rigid Control of Eating Scale (FORCES)

**Esnek veya Katı Yeme Kontrolü Ölçeği (EKYKÖ)**	**Kesinlikle doğru değil**	**Doğru değil**	**Doğru**	**Kesinlikle doğru**
**1.** Ne kadar yiyebileceğim konusunda katı kurallarım vardır.				
**2.** Ne yediğime çok dikkat ederim.				
**3.** Diyetime uyduğumdan emin olmak için çok zaman harcarım.				
**4.** Yediklerimin besin içeriğini kontrol ederim.				
**5.** Yediklerimin ayrıntılı kaydını tutarım.				
**6.** Normalde yememe izin vermeyeceğim bir şeyi yediğimde kendimi başarısız hissederim.				
**7.** Normalde yememe izin vermeyeceğim bir şeyi yediğimde kendime kızarım.				
**8.** Normalde yememe izin vermeyeceğim bir şeyi yediğimde kendimi hayal kırıklığına uğramış hissederim.				
**9.** Normalde yememe izin vermeyeceğim bir şeyi yediğimde, kendimi üzgün hissederim.				
**10.** Normalde yememe izin vermeyeceğim bir şeyi yediğimde kendimi gergin, sinirli ve/veya depresif hissederim.				
**11.** Ne kadar yediğim konusunda kontrolümü kaybedeceğimden endişelenirim.				
**12.** Ne yediğim konusunda kontrolümü kaybedeceğimden endişelenirim.				
**13.** Ben yiyecekleri değil, sanki yiyecekler beni kontrol ediyormuş gibi hissederim.				
**14.** Herhangi bir yiyeceğin ara sıra yenmesinin uygun olduğunu düşünüyorum.				
**15.** Herhangi bir yiyeceğin az miktarda yenmesinin uygun olduğunu düşünüyorum.				
**16.** Normalde yememe izin vermeyeceğim yiyecekleri bazen yemenin sorun olmayacağını düşünürüm.				
**17.** Normalde yememe izin vermeyeceğim bir şeyi yediğimde kendimi çok keyifli hissederim.				
**18.** Normalde yememe izin vermeyeceğim bir şeyi yediğimde zevk alırım.				
**19.** Normalde yememe izin vermeyeceğim bir şeyi yediğimde memnun hissederim.				
