# Supplementary material for: Psychometric properties of the Turkish version of the Flexible or Rigid Control of Eating Scale in women
Source: Front Psychol. 2026 Sep 17;17:1826677. doi: 10.3389/fpsyg.2026.1826677 (PMC13627051; doi:10.3389/fpsyg.2026.1826677)
Supplement: Supplementary file 3 [file Table_2.pdf]

Supplementary Table S2. Infit and Outfit Mean Square (MnSq) Statistics for FORCES Subscales

FORCES	Subscale	Item	Measure	SE	Infit	Outfit	DIF for BMI (adj. p)
Rigid control	Strict Behaviors	Item 1	-2.00	0.0831	1.064	1.057	**<0.001**
		Item 2	-.2.92	0.0793	1.111	1.153	0.268
		Item 3	-.1.83	0.0841	0.887	0.867	0.109
		Item 4	-2.86	0.0795	1.248	1.255	0.732
		Item 5	-1.20	0.0878	1.066	1.006	**0.036**
	Negative Emotions	Item 6	-2.54	0.0807	0.893	0.867	0.396
		Item 7	-2.74	0.0799	0.887	0.880	0.770
		Item 8	-2.75	0.0799	0.811	0.791	0.113
		Item 9	-2.79	0.0797	0.852	0.844	0.170
		Item 10	-2.31	0.0817	0.900	0.863	0.998
	Worry	Item 11	-2.28	0.0818	0.974	0.954	0.580
		Item 12	-2.28	0.0818	0.922	0.894	NaN
		Item 13	-1.99	0.0832	1.418	1.424	NaN
Flexible control	Flexible Beliefs	Item 14	-3.28	0.0828	1.158	1.144	0.548
		Item 15	-2.94	0.0820	1.113	1.129	**0.031**
		Item 16	-.2.91	0.0820	0.917	0.903	0.934
	Positive Emotions	Item 17	-2.01	0.0812	0.929	0.930	0.414
		Item 18	-.1.98	0.0812	0.886	0.906	0.419
		Item 19	-1.86	0.0813	0.976	0.971	0.066

NaN: Not calculable due to insufficient category frequencies in the obese subgroup.
