# Supplementary material for: Psychometric properties of the Turkish version of the Flexible or Rigid Control of Eating Scale in women
Source: Front Psychol. 2026 Sep 17;17:1826677. doi: 10.3389/fpsyg.2026.1826677 (PMC13627051; doi:10.3389/fpsyg.2026.1826677)
Supplement: Supplementary file 4 [file Table_3.pdf]

Supplementary Table S3. Rasch model fit summary for FORCES subfactor

Factor	Person Reliability	MADaQ3	p
Rigid control	0.901	0.228	**< 0.001**
Flexible control	0.807	0.370	**< 0.001**

*Lower MADaQ3 indicates better local independence, **FORCES:** The Flexible or Rigid Control of Eating Scale.
