# Supplementary material for: Psychometric properties of the Turkish version of the Flexible or Rigid Control of Eating Scale in women
Source: Front Psychol. 2026 Sep 17;17:1826677. doi: 10.3389/fpsyg.2026.1826677 (PMC13627051; doi:10.3389/fpsyg.2026.1826677)
Supplement: Supplementary file 5 [file Table_4.pdf]

Supplementary Table S4. CFA Standardized Loadings, Average Variance Extracted, and Composite Reliability

Factor	Items	Standardized CFA Loadings (λ)	AVE	CR
	Item 1	0.732		
	Item 2	0.788		
**Strict Behaviors**	Item 3	0.667	**0.502**	**0.834**
	Item 4	0.693		
	Item 5	0.654		
	Item 6	0.828		
	Item 7	0.835		
**Negative Emotions**	Item 8	0.924	**0.725**	**0.929**
	Item 9	0.870		
	Item 10	0.796		
	Item 11	0.842		
**Worry**	Item 12	0.884	**0.666**	**0.856**
	Item 13	0.712		
	Item 14	0.758		
**Flexible Beliefs**	Item 15	0.840	**0.571**	**0.798**
	Item 16	0.659		
	Item 17	0.807		
**Positive Emotions**	Item 18	0.911	**0.743**	**0.896**
	Item 19	0.864		

AVE: Average Variance Extracted, **CR:** Composite Reliability. **Note:** AVE and CR were calculated using standardized factor loadings and error variances obtained from the first-order CFA model in Study 2. AVE values > 0.50 and CR values > 0.70 indicate satisfactory convergent validity and composite reliability.
